# A Rare Case of Hemophagocytic Lymphohistiocytosis Triggered by Intravesical Bacillus Calmette-Guérin (BCG) for Bladder Cancer

**DOI:** 10.7759/cureus.96711

**Published:** 2025-11-12

**Authors:** Mizuki Nakayama, Toyoshi Yanagihara, Makoto Fujimoto, Natsumi Kushima, Yu Okabe, Mikiko Aoki, Kaori Koga, Keiji Yokoyama, Yuka Sakaki, Takato Ikeda, Naoki Hamada, Makoto Hamasaki, Masaki Fujita

**Affiliations:** 1 Department of Respiratory Medicine, Fukuoka University Hospital, Fukuoka, JPN; 2 Department of Urology, Fukuoka University Hospital, Fukuoka, JPN; 3 Department of Pathology, Fukuoka University Hospital, Fukuoka, JPN; 4 Department of Gastroenterology and Medicine, Fukuoka University Hospital, Fukuoka, JPN

**Keywords:** hemophagocytic lymphohistiocytosis (hlh)

## Abstract

Intravesical bacillus Calmette-Guérin (BCG) therapy is widely used for non-muscle-invasive bladder cancer but can rarely lead to severe systemic complications. Hemophagocytic lymphohistiocytosis (HLH) is a life-threatening hyperinflammatory syndrome characterized by fever, cytopenias, and organ dysfunction. We describe a 63-year-old man who developed HLH after re-induction BCG therapy. He presented with fever, hepatosplenomegaly, pancytopenia, and liver and kidney dysfunction. Laboratory tests revealed hyperferritinemia and elevated soluble interleukin-2 receptor levels. Liver biopsy showed non-caseating granulomas without acid-fast bacilli, and bone marrow biopsy confirmed hemophagocytosis. Urine culture yielded Mycobacterium tuberculosis complex, consistent with prior BCG instillation. Treatment with high-dose corticosteroids, intravenous immunoglobulin, and BCG-active antibiotics resulted in rapid recovery. This case illustrates that HLH is a rare but serious complication of intravesical BCG, and emphasizes the importance of early recognition and combined immunosuppressive and anti-mycobacterial therapy.

## Introduction

Intravesical bacillus Calmette-Guérin (BCG) is a first-line adjuvant immunotherapy for non-muscle-invasive bladder cancer [1]. It works by provoking a local antitumor immune response in the bladder. Most patients experience only self-limited local symptoms such as dysuria, frequency, low-grade fever, malaise, or cystitis [1]. Serious adverse events are uncommon (~1-5%). Among them, systemic complications are rare (around ~1% or less) and include pneumonitis, hepatitis, sepsis-like illness, and a systemic inflammatory reaction sometimes termed “BCGitis” [2,3]. These events typically arise after several instillations rather than the first dose.

Hemophagocytic lymphohistiocytosis (HLH), previously known as hemophagocytic syndrome, is a hyperinflammatory syndrome caused by uncontrolled activation of macrophages and T cells with excessive cytokine release [4]. Secondary HLH can be triggered by infections (including mycobacterial disease), malignancy, or drugs, and tuberculosis-associated HLH carries high mortality if treatment is delayed [5]. HLH after intravesical BCG is exceedingly rare, with only scattered case reports [6-11]. Here, we report a patient who developed HLH after re-induction BCG, illustrating the need for early recognition and coordinated treatment.

## Case presentation

A 63-year-old man was referred with gross hematuria six months before admission. Cystoscopy and transurethral resection confirmed bladder cancer. Pathology showed urothelial carcinoma in situ (pTis, G3) and an invasive urothelial carcinoma with squamous differentiation (pTx, G3), with an intermediate invasive growth pattern at the tumor invasive front (INFb) and no lymphovascular invasion (LVI0). He underwent transurethral resection of the bladder tumor (TURBT) and, five months before admission, completed an induction course of intravesical BCG given weekly for six instillations. Three months before admission, surveillance cystoscopy revealed a papillary tumor at the ureteral orifice, consistent with BCG failure and suspected extension into the left ureter. Repeat transurethral resection with ureteral biopsies found no definite malignancy, but urine cytology was Papanicolaou class V; a ureteral stent was placed for left ureteral stricture. Re-induction BCG was started six weeks before admission. He had local bladder symptoms (dysuria, hematuria, minor leakage) without major adverse events through four instillations. After the fifth instillation (three weeks before admission), symptoms worsened and the sixth planned instillation (two weeks before admission) was postponed. One week before admission, he developed anorexia and fatigue; outpatient tests showed liver and kidney dysfunction and hyponatremia, prompting emergency admission.

**Figure 1:**
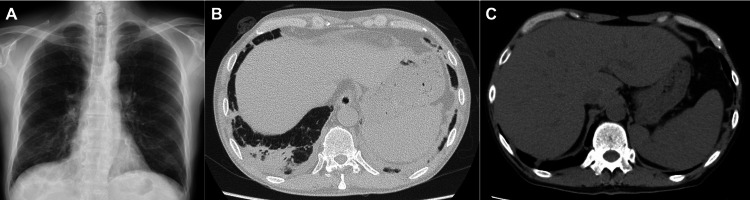
Findings at Initial Presentation (A) Chest radiograph shows no obvious abnormalities. (B) Chest CT demonstrates consolidation in the right lower lobe adjacent to the pleura. (C) Abdominal CT reveals hepatosplenomegaly without gallbladder enlargement or wall thickening.

On admission, he was alert and febrile to 38.2 °C; blood pressure 105/55 mmHg, heart rate 92/min, respiratory rate 16/min, and oxygen saturation 94% on room air. Examination showed mild scleral icterus; lungs were clear and the abdomen was soft and non-tender. The liver edge was palpable below the right costal margin, and the spleen was not palpable. Initial laboratories revealed a WBC count of 3.8 × 10^3/µL and mild thrombocytopenia (111 × 10^3/µL), with a normal hemoglobin level (13.2 g/dL) (Table 1). Liver and kidney injury were present (aspartate aminotransferase (AST) 271 U/L, alanine aminotransferase (ALT) 212 U/L, γ-glutamyltransferase 335 U/L, alkaline phosphatase 1,097 U/L; creatinine 1.75 mg/dL). Inflammatory markers were elevated (C-reactive protein 2.49 mg/dL; ferritin 4,439 ng/mL; soluble interleukin (IL)-2 receptor 8,894 U/mL) (Table 1). Viral serologies were negative. Chest radiograph was unremarkable (Figure 1A). CT scan showed lower-lobe-predominant consolidations and hepatosplenomegaly (Figure 1B, 1C). Abdominal ultrasound confirmed hepatosplenomegaly but no structural biliary abnormality to explain the cholestatic enzyme elevation.

**Table 1:** Laboratory findings of the patient. Comprehensive laboratory test results are summarized with corresponding reference ranges for interpretation. Abbreviations: WBC, white blood cell; RBC, red blood cell; Hb, hemoglobin; Plt, platelet; TP, total protein; Alb, albumin; CRP, C-reactive protein; AST, aspartate aminotransferase; ALT, alanine aminotransferase; LD, lactate dehydrogenase; γ-GTP, gamma-glutamyl transferase; ALP, alkaline phosphatase; T-Bil, total bilirubin; BUN, blood urea nitrogen; TG, triglyceride, Cr, creatinine; Na, sodium; K, potassium; Ig, immunoglobulin; sIL-2R, soluble interleukin-2 receptor; ANA, antinuclear antibody; CMV-IgM, cytomegalovirus immunoglobulin M; EBCA-IgM, Epstein–Barr capsid antigen immunoglobulin M; HEV-IgA, hepatitis E virus immunoglobulin A; MitM2-Ab, anti-mitochondrial M2 antibody.

Test	Value	Reference range	Test	Value	Reference range
WBC (/uL)	3800	3,300-8,600	TG (mg/dL)	143	40-234
RBC (10^4/μL)	448	435-555	Cr (mg/dL)	1.75	0.65-1.07
Hb (g/dL)	13.2	13.7-16.8	Na (mEq/L)	124	138-145
Plt (10^3/uL)	111	158-348	K (mEq/L)	5.8	3.6-4.8
TP(g/dL)	6.0	6.6-8.1	IgG (mg/dL)	1082	861-1747
Alb(g/dL)	2.8	3.8-5.3	IgA (mg/dL)	143	93-393
CRP (mg/dL)	2.49	0.4-1.5	IgM (mg/dL)	40	50-269
AST (U/L)	271	13-30	sIL-2R (U/mL)	8894	121-613
ALT (U/L)	212	10-42	Ferritin (ng/mL)	4439	40-465
LD (U/L)	501	124-222	ANA	<40	<40
γ-GTP (U/L)	335	13-64	CMV-IgM	0.17	<0.85
ALP (U/L)	1097	38-113	EBCA-IgM	<10	<10
T-Bil (mg/dL)	1.4	0.4-1.5	HEV-IgA	negative	negative
BUN (mg/dL)	27	8-20	MitM2-Ab	negative	negative

**Figure 2:**
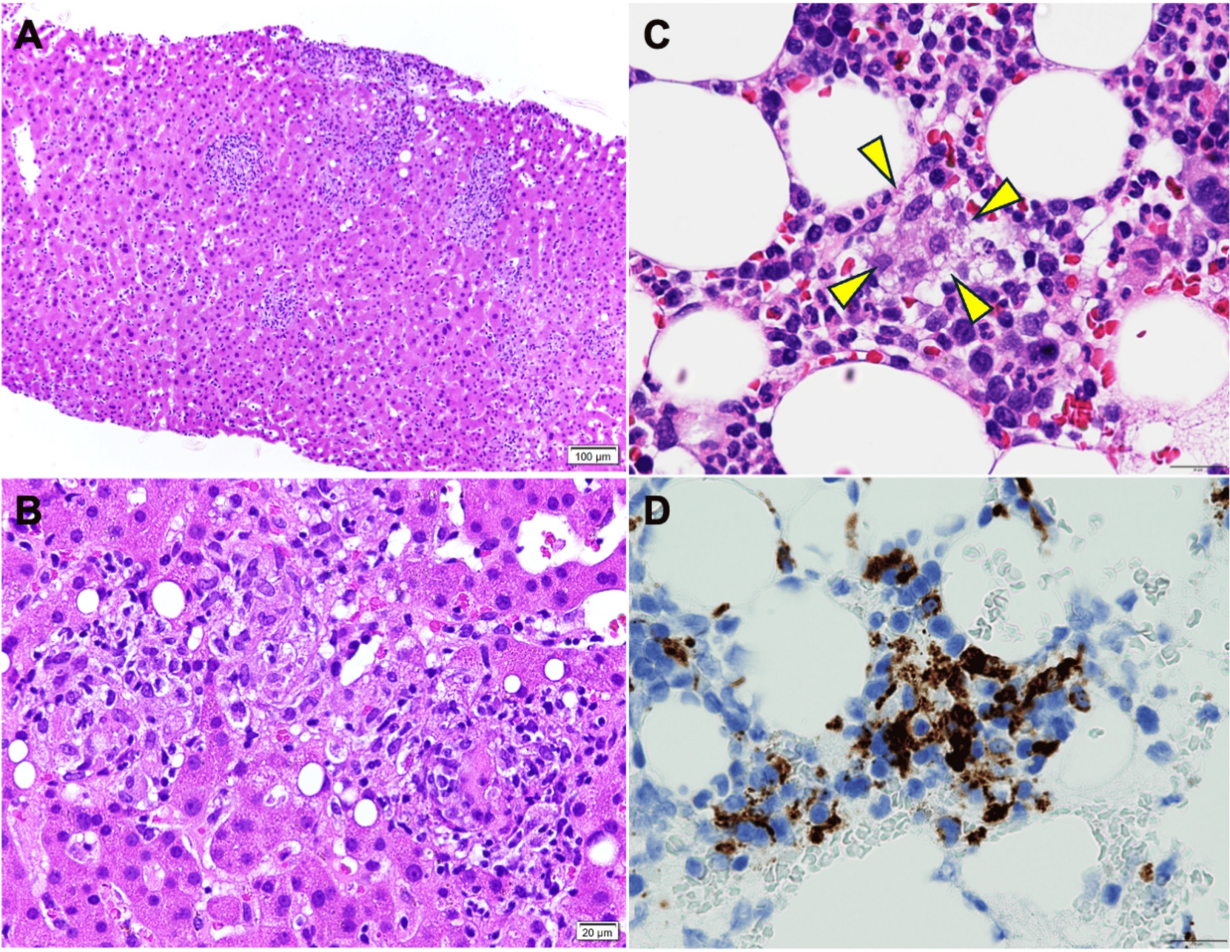
Histopathological findings of liver and bone marrow biopsies. (A) Liver biopsy showing epithelioid cell granulomas with surrounding lymphocytic infiltration (hematoxylin and eosin [H&E], scale bar = 100 μm). (B) Higher magnification of the liver specimen demonstrating well-formed non-caseating granulomas (H&E, scale bar = 20 μm). (C) H&E staining of bone marrow biopsy showing hemophagocytosis (arrowheads). (D) CD68 immunohistochemistry indicating hemophagocytosis by CD68-positive histiocytes (scale bars = 20 μm).

On day eight, a urine transcription-reverse transcription concerted (TRC) assay for Mycobacterium tuberculosis complex was positive; given concern for disseminated BCG, rifampicin, isoniazid, and ethambutol were started, and urine mycobacterial culture later grew Mycobacterium tuberculosis complex. Repeated respiratory samples remained negative. On day 10, liver biopsy showed epithelioid granulomas with lymphocytic infiltration (Figure 2A, 2B); Ziehl-Neelsen staining was negative for acid-fast bacilli (AFB). The white blood cell count fell to 1,600/µL on day 10; granulocyte colony-stimulating factor was given for two days with transient recovery, followed by recurrent leukopenia, and fevers >38 °C persisted.

By day 22, anemia had developed (hemoglobin 8.8 g/dL). He met the HLH-2024 clinical pathway at treatment initiation, fulfilling five of seven items (fever, splenomegaly on CT, ≥2-lineage cytopenias, hyperferritinemia 4,439 ng/mL, and elevated soluble IL-2 receptor 8,894 U/mL) [12]. Bone marrow later confirmed hemophagocytosis without malignant infiltration (Figure 2C, 2D), raising the total to six of seven; hypertriglyceridemia/hypofibrinogenemia was not met. Key differentials were systematically assessed. Sepsis and malignancy-associated HLH were unlikely based on negative cultures, absence of an imaging source, and bone marrow showing hemophagocytosis without malignant infiltration.

**Figure 3:**
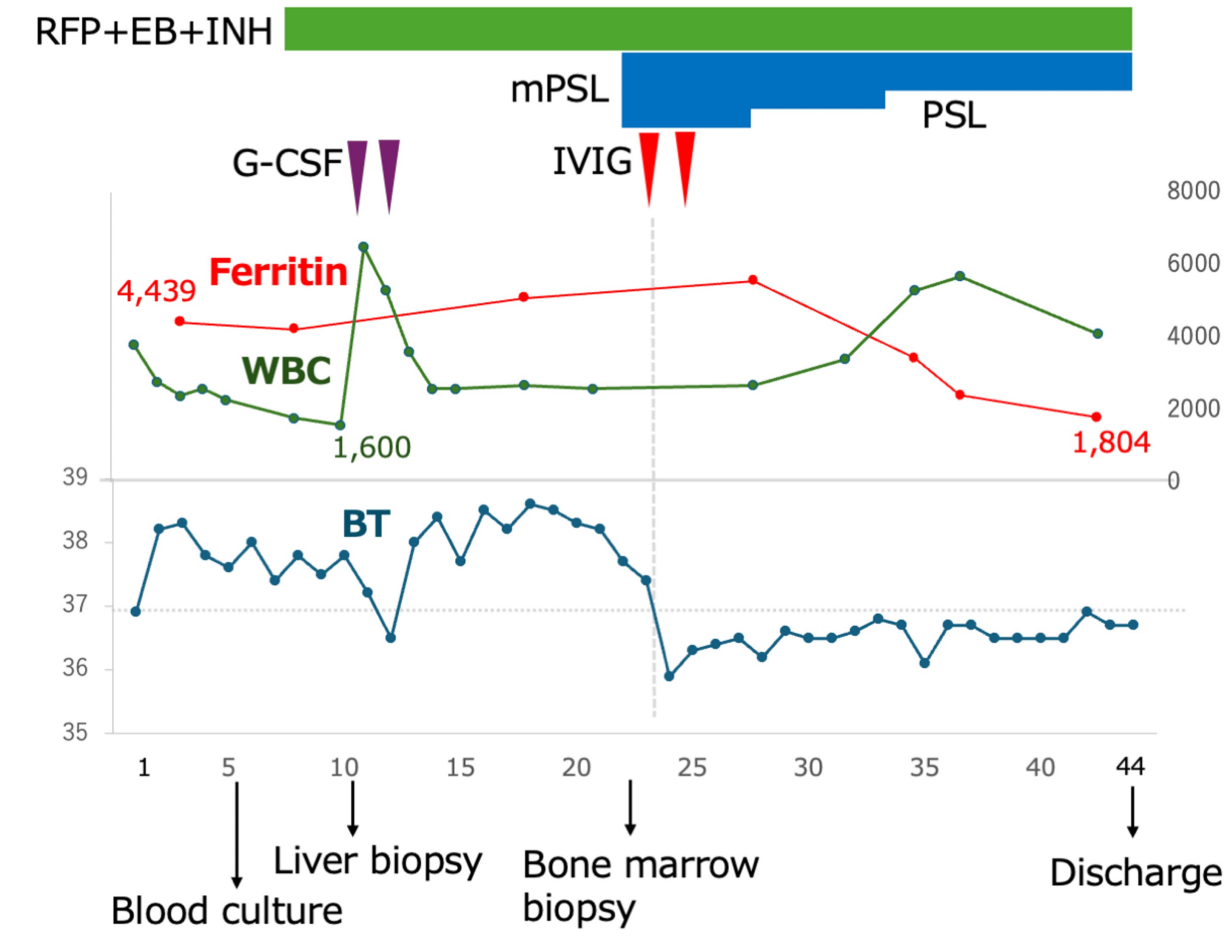
Clinical course of the patient. Clinical course of the patient. White blood cell (WBC) counts (green line), serum ferritin levels (red line), and body temperature (BT, blue line) are shown from admission (day 1) to discharge (day 44). Treatments and interventions are indicated above the graph, including anti-tuberculous therapy with rifampicin, ethambutol, and isoniazid (RFP + EB + INH), granulocyte colony-stimulating factor (G-CSF), intravenous immunoglobulin (IVIG), and corticosteroids (methylprednisolone [mPSL], prednisolone [PSL]). Timing of blood culture, liver biopsy, and bone marrow biopsy is shown below the x-axis. Clinical improvement was observed following initiation of IVIG and corticosteroids, with resolution of fever, recovery of blood counts, and decline in ferritin levels.

On day 23, intravenous immunoglobulin (IVIG) (20 g/day for two days) and methylprednisolone (mPSL; 125 mg IV) were initiated, resulting in rapid resolution of fever, recovery of blood cell counts, a decline in ferritin, and improvement in hepatosplenomegaly (Figure 3). The steroid regimen was mPSL 125 mg IV daily for five days, then 62.5 mg IV daily for two days, followed by an oral prednisolone (PSL) taper (80 mg/day for five days, 60 mg/day for four days, 50 mg/day for eight days, and 40 mg/day at discharge). Because rifampicin induces hepatic enzymes and reduces glucocorticoid exposure, we administered approximately twice the anticipated steroid dose. He was discharged in improved condition on day 44.

## Discussion

We present the case of HLH occurring after re-induction of intravesical BCG therapy. Two mechanisms are thought to explain BCG-related HLH: (1) disseminated Mycobacterium bovis infection and (2) a hyperinflammatory, immune-mediated response without viable organisms. In previously published cases, disseminated M. bovis was confirmed only rarely, suggesting that HLH may more often arise as an immune-mediated hyperinflammatory reaction rather than true dissemination [8,9]. Non-caseating granulomas with negative stains and cultures support a hypersensitivity phenotype, whereas demonstration of viable M. bovis in tissue supports disseminated infection. In practice, diagnosis must be made clinically and integratively, since AFB smear, culture, and molecular assays are only modestly sensitive. Our patient developed HLH after re-induction BCG and showed granulomatous hepatitis with negative AFB stains in liver and bone marrow and negative blood cultures, favoring a hypersensitivity-dominant phenotype. Mechanistically, we speculate that intravesical BCG engages pattern-recognition receptors on monocytes and macrophages, inducing production of IL-1β, IL-6, TNF-α, and IL-12 and promoting a Th1-skewed response with excess IFN-γ [13]. IFN-γ amplifies macrophage activation and hemophagocytosis [14], and T-cell activation is reflected by elevated soluble interleukin-2 receptor levels. BCG re-induction in this case may also evoke “trained immunity” in innate cells, lowering the threshold for a hyperinflammatory flare.

In this case, CT scan imaging showed lower-lobe-predominant consolidations. Although BCG pneumonitis could not be ruled out, the subpleural right lower-lobe consolidation was considered most compatible with HLH-associated pneumonitis, given the negative result of TRC for M. tuberculosis, systemic hyperinflammation, and the clinical response following IVIG and corticosteroids.

Recent advances in diagnostic criteria are also relevant. In 2024, the Histiocyte Society revised the HLH-2004 framework, publishing updated HLH-2024 criteria [12]. These incorporate two complementary diagnostic pathways: a clinical pathway requiring fulfillment of five of seven criteria, and a genetic or cellular cytotoxicity pathway primarily for familial HLH. The revision confirmed the ongoing utility of the HLH-2004 criteria, especially in settings where NK-cell function testing is not available, while improving sensitivity and specificity for pediatric and familial HLH. Although designed mainly for primary HLH, these updates are likely to influence the diagnostic approach in secondary and adult HLH, including BCG-associated cases such as ours.

Management requires rapid control of hyperinflammation together with coverage for possible BCG infection. Most reported cases have been treated with high-dose corticosteroids, intravenous immunoglobulin, and triple anti-tuberculous therapy (rifampicin, isoniazid, and ethambutol) [6-11]. Because M. bovis is intrinsically resistant to pyrazinamide, step-down to rifampicin and isoniazid after two months is commonly practiced. When liver or kidney dysfunction is present, very early initiation of anti-mycobacterial therapy before immunomodulation may result in treatment interruption or dose adjustments; in such cases, starting corticosteroids and IVIG first may be safer if HLH is promptly diagnosed. Immunosuppression should be tailored to severity. High-dose glucocorticoids (e.g., methylprednisolone 2-2.5 mg/kg/day or dexamethasone 10 mg) with or without IVIG are first-line, while etoposide is reserved for progressive or organ-threatening disease and may be withheld when hepatic or renal impairment is marked. These strategies are consistent with both published BCG-HLH cases and current practice in adult HLH. Regarding duration, prior BCG-associated HLH reports rarely provide detailed steroid tapers. In our case, the steroid tapers were individualized to clinical response and to organ function.

Reported outcomes appear more favorable in BCG-associated HLH compared with tuberculosis-associated HLH overall. While tuberculosis-HLH carries a mortality of 45-50%, nearly all published cases of BCG-HLH have recovered with timely administration of steroids/IVIG and BCG-active therapy. This underscores the importance of early recognition and decisive management.

Two practical lessons should be emphasized. First, severe systemic reactions such as HLH should prompt permanent discontinuation of intravesical BCG to prevent relapse. Second, HLH may be complicated by thrombosis, as highlighted by a reported case of concurrent pulmonary embolism; thrombo-inflammatory mechanisms such as neutrophil extracellular traps have been proposed, warranting vigilance for venous thromboembolism.

Finally, this case illustrates diagnostic pitfalls. Urine M. tuberculosis complex positivity after intravesical BCG does not prove hematogenous dissemination. Histology, imaging, and multi-site microbiological testing must be interpreted together, keeping in mind the limited sensitivity of mycobacterial assays. In any patient with post-BCG fever, cytopenias, and hyperferritinemia, clinicians should screen for HLH, obtain bone marrow and (when feasible) liver tissue, initiate high-dose steroids ± IVIG, and start BCG-active antibiotics, with treatment modified according to the patient’s organ function.

## Conclusions

HLH is a rare but serious complication of intravesical BCG therapy. Early recognition and combined treatment with immunosuppression and BCG-active antibiotics are crucial for favorable outcomes.

## References

[1] Sylvester RJ, van der Meijden AP, Witjes JA, Kurth K (2005). Bacillus calmette-guerin versus chemotherapy for the intravesical treatment of patients with carcinoma in situ of the bladder: a meta-analysis of the published results of randomized clinical trials. J Urol.

[2] Khalili N, Mohammadzadeh I, Khalili N, Heredia RJ, Zoghi S, Boztug K, Rezaei N (2021). BCGitis as the primary manifestation of chronic granulomatous disease. IDCases.

[3] Yong C, Steinberg RL, O'Donnell MA (2020). Severe infectious complications of intravesical Bacillus Calmette-Guérin: a case series of 10 patients. Urology.

[4] Henter JI (2025). Hemophagocytic lymphohistiocytosis. N Engl J Med.

[5] Fauchald T, Blomberg B, Reikvam H (2023). Tuberculosis-associated hemophagocytic lymphohistiocytosis: a review of current literature. J Clin Med.

[6] Schleinitz N, Bernit E, Harle JR (2002). Severe hemophagocytic syndrome after intravesical BCG instillation. Am J Med.

[7] Misra S, Gupta A, Symes A, Duncan J (2014). Haemophagocytic syndrome after intravesical bacille Calmette-Guérin instillation. Scand J Urol.

[8] Manganas K, Angelara M, Bountzona I, Karamanakos G, Toskas A (2022). Secondary haemophagocytic lymphohistiocytosis syndrome (HLH) after intravesical instillation of Bacillus Calmette-Guérin (BCG): a case report and review of the literature. Eur J Case Rep Intern Med.

[9] Liatsos GD, Manousopoulou G, Poulaki A, Iliaki A, Mariolis I, Vassilopoulos D (2023). Haemophagocytic lymphohistiocytosis after intravesical BCG administration for bladder cancer presenting with multiorgan failure. Access Microbiol.

[10] Li TL, Huang CP, Lin CY, Ho MW, Cho CH, Chen YH, Chen WC (2024). Hemophagocytic syndrome in a patient of upper urinary tract urothelial cancer after Bacillus Calmette-Guérin instillation: a case report. Urol Case Rep.

[11] Fabozzi A, Paciucci G, Repaci E (2024). Hemophagocytic lymphohistiocytosis and pulmonary embolism caused by bacillus Calmette-Guerin intravesical instillation. Acta Biomed.

[12] La Rosée P, La Rosée F (2024). HLH: diagnostics revisited and improved. Blood.

[13] Heldwein KA, Liang MD, Andresen TK (2003). TLR2 and TLR4 serve distinct roles in the host immune response against Mycobacterium bovis BCG. J Leukoc Biol.

[14] Locatelli F, Jordan MB, Allen C (2020). Emapalumab in children with primary hemophagocytic lymphohistiocytosis. N Engl J Med.

